# Combination of Polygoni Multiflori Radix Praeparata and Acori Tatarinowii Rhizoma Alleviates Learning and Memory Impairment in Scopolamine-Treated Mice by Regulating Synaptic-Related Proteins

**DOI:** 10.3389/fphar.2021.679573

**Published:** 2021-07-29

**Authors:** Funan Ning, Lvyi Chen, Linlin Chen, Xin Liu, Yao Zhu, Jiayi Hu, Guangjing Xie, Jiaxuan Xia, Kun Shi, Zhou Lan, Ping Wang

**Affiliations:** ^1^ School of Pharmacy, Hubei University of Chinese Medicine, Wuhan, China; ^2^ Department of Pharmacy, Hubei Provincial Hospital of Integrated Chinese and Western Medicine, Wuhan, China; ^3^ School of Pharmacy, South-Central University for Nationalities, Wuhan, China; ^4^ School of Basic Medicine, Hubei University of Chinese Medicine, Wuhan, China

**Keywords:** Acori Tatarinowii Rhizoma, scopolamine, learning and memory, Alzheimer’s disease, Polygoni Multiflori Radix Praeparata

## Abstract

Polygoni Multiflori Radix Praeparata (ZhiHeShouWu, PMRP) and Acori Tatarinowii Rhizoma (ShiChangPu, ATR) and their traditional combination (PA) are frequently used in traditional Chinese medicine to prevent and treat Alzheimer disease (AD) based on the theory that PMRP tonifies the kidney and ATR dissipates phlegm. However, the components of PA and their mechanisms of action are not known. The present study analyzed the active components of PA, and investigated the protective effect of PA against cognitive impairment induced by scopolamine in mice along with the underlying mechanism.The aqueous extract of PA was analyzed by high-performance liquid chromatography–mass spectrometry (HPLC-MS) and gas chromatography (GC)-MS in order to identify the major components. To evaluate the protective effect of PA against cognitive dysfunction, mice were orally administered PA, PMRP, or ATR for 30 days before treatment with scopolamine. Learning and memory were assessed in mice with the Morris water maze test; neurotransmitter levels in the hippocampus were analyzed by HPLC-MS; and the expression of synapse-related proteins in the hippocampus was detected by western blotting and immunohistochemistry. Eight active compounds in PA and rat plasma were identified by HPLC-MS and GC-MS. Plasma concentrations of 2,3,5,4′-tetrahydroxystilbene-2-O-β-d-glucoside, emodin, α-asarone, and asarylaldehyde were increased following PA administration; meanwhile, gallic acid, emodin-8-O-β-d-glucopyranoside, β-asarone, and *cis*-methyl isoeugenol concentrations were similar in rats treated with PA, PMRP, and ATR. In scopolamine-treated mice, PA increased the concentrations of neurotransmitters in the hippocampus, activated the brain-derived neurotrophic factor (BDNF)/extracellular signal-regulated kinase (ERK)/cAMP response element binding protein (CREB) signaling pathway, and increased the expression of p90 ribosomal S6 kinase (p90RSK) and postsynaptic density (PSD)95 proteins. Thus, PA alleviates cognitive deficits by enhancing synaptic-related proteins, suggesting that it has therapeutic potential for the treatment of aging-related diseases such as AD.

## Introduction

Most aging-associated neurodegenerative disorders, including Alzheimer disease (AD) are characterized by progressive memory loss and learning deficits. The cholinergic hypothesis has been proposed to explain the pathogenesis of AD Terry and Buccafusco (2003), Craig et al. (2011) based on the observation that AD patients exhibit a marked loss of cortical cholinergic innervation in the cortex and hippocampus, with corresponding cognitive deficits (Savonenko et al., 2012). This is associated with the production of the toxic form of amyloid β (Aβ) protein, which attenuates cholinergic signaling and causes damage to cholinergic neurons (Hellström-Lindahl, 2000; Kar et al., 2004; Williams et al., 2006). Cholinergic neurotransmission enhances afferent inputs and synapses and contributes to the encoding of novel information in brain areas related to memory (Schliebs and Arendt 2011; Weon et al., 2016). The level of acetylcholine (ACh) in neuronal synapses is maintained by acetylcholinesterase (AChE).

The main drugs used for AD treatment are AChE inhibitors such as donepezil, galantamine, and tacrine, which reduce extrasynaptic metabolism of ACh and thereby enhance its concentration at the synaptic cleft Ionita et al. (2018); and N-methyl-d-aspartic acid glutamate receptor antagonists such as memantine (Tayeb et al., 2012). However, these drugs only slow the deterioration of cognitive function and do not promote neuronal survival; moreover, they have various adverse effects including nausea, diarrhea, and vomiting. As such, there is a need for novel agents for AD treatment that are better tolerated but also effective.

Traditional Chinese medicines (TCMs) have certain advantages over conventional drugs for the treatment of AD, as herbal formulations typically have numerous components that can act on multiple targets. Polygoni Multiflori Radix Praeparata (ZhiHeShouWu, PMRP) is the dried root of *Polygonum multiflorum* Thunb. prepared with fermented black bean liquid. PMRP is warm, with a sweet, bitter and sharp taste, and can invigorate the liver and kidney, enhance blood, darken hair, and increase bone and muscle strength (Chinese Pharmacopoeia Commission, 2015). PMRP has been shown to exert anti-aging and anti-inflammatory effects and enhance immune function (Mei et al., 2016). Acori Tatarinowii Rhizoma (ShiChangPu, ATR) is the dried rhizome of *Acorus tatarinowii* Schott. Like PMRP, ATR is warm with an acrid and bitter taste; it is known to dissipate phlegm and can be used for resuscitation and improving the mind (Chinese Pharmacopoeia Commission, 2015). ATR was also shown to promote neural progenitor proliferation, improve memory and cognitive function, and protect against Aβ-induced neurotoxicity (An et al., 2014; Deng et al., 2015; Mao et al., 2015).

In China, the traditional combination of PMRP and ATR (PA) is often used to achieve a synergistic effect in the treatment of aging-related diseases. Studies have shown that drug pair PMRP and ATR is most frequently used to prevent AD based on invigorating kidney and resolving phlegm. The representative prescription for the treatment of AD with this drug pair is the Yangshou Dan, Huan Nao Yi Cong Formula, etc., recorded in “Royal Pharmacy prescriptions.” The compatibility of PA can nourish the kidney and intelligence, and resolve phlegm that fit the mechanism of AD kidney deficiency and phlegm blocking in Chinese medicine (Xing et al., 2014; Du et al., 2017; Yang et al., 2019). However, the molecular basis of PA’s effects has not been elucidated. In the present study, we analyzed the active components of PA by high-performance liquid chromatography-mass spectrometry (HPLC-MS) and gas chromatography (GC)-MS and investigated the protective effect of PA against cognitive impairment induced by scopolamine in mice as well as the possible underlying mechanism.

## Materials and Methods

### Reagents

**FIGURE 1:**
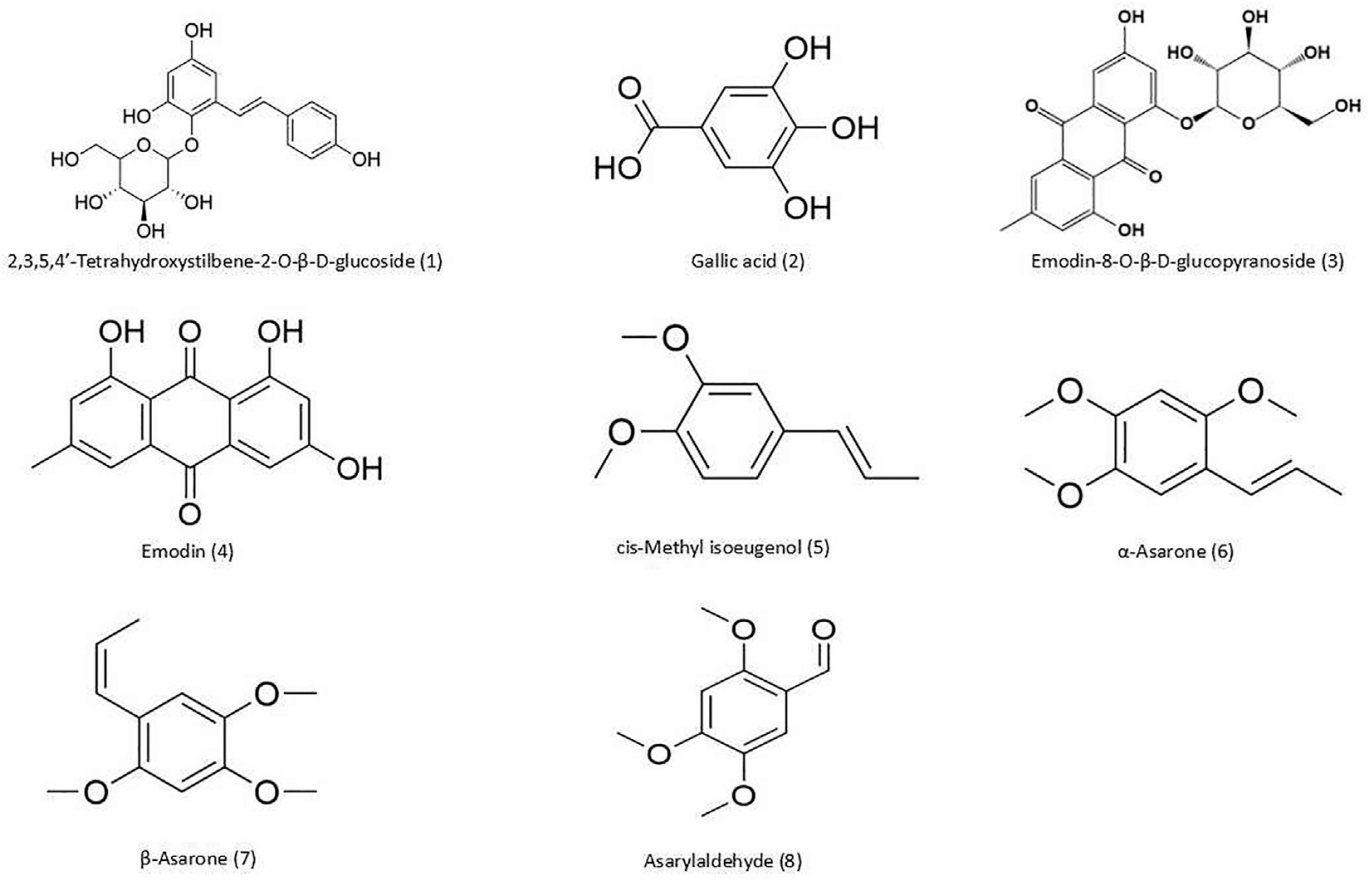
Chemical structures of compounds detected in PA. (1) THSG; (2) gallic acid; (3) emodin-8-O-β-d-glucopyranoside; (4) emodin; (5) *cis*-methyl isoeugenol; (6) α-asarone; (7) β-asarone; and (8) asarylaldehyde.

The raw materials of PMRP and ATR were purchased from Hubei Jurui Chinese Medicine Decoction Pieces Co. (Wuhan, China; batch no. 171101) and Hubei Gongshengtang Chinese Medicine Pieces Co. (Wuhan, China; batch no. 170901). 2,3,5,4′-Tetrahydroxystilbene-2-O-β-D-glucoside (THSG; C_20_H_22_O_9_, 98% purity), gallic acid (C_7_H_6_O_5_, 98% purity), emodin-8-O-β-D-glucopyranoside (C_21_H_20_O_10_, 98% purity), emodin (C_15_H_10_O_5_, 98% purity), β-asarone (C_12_H_16_O_3_, 98% purity), α-asarone (C_12_H_16_O_3_, 98% purity), *cis*-methyl isoeugenol (C_11_H_14_O_2_, 98% purity), asarylaldehyde (C_10_H_12_O_4_, 98% purity), ibuprofen (C_13_H_18_O_2_), and naphthalene (C_10_H_8_) were from Beijing Zhongke Yingchuang Biotechnology Co. (Beijing, China). The structures of these compounds are shown in Figure 1. Methanol and acetonitrile [mass spectrometry (MS) grade] were from Thermo Fisher Scientific (Waltham, MA, United States). Scopolamine Hydrobromide was from Sigma-Aldrich (St. Louis, MO, United States). Rabbit antibodies against brain-derived neurotrophic factor (BDNF; catalog no. A11028), tyrosine receptor kinase B (TrkB; catalog no. A12325), extracellular signal-regulated protein kinase (ERK; catalog no. A16686), phosphorylated (p-) ERK (catalog no. AP0472), cyclic AMP response element-binding protein (CREB; catalog no. A2431), and *p*-CREB (catalog no. AP0333) were from ABclonal (Boston, MA, United States); and actin (GB12001), antibodies against p90 ribosomal S6 kinase (p90RSK; catalog no. A15718) and postsynaptic density (PSD)-95 (catalog no. A6194) were from Wuhan Aibotech Biotechnology Co. (Wuhan, China).

### Animals

Specific pathogen-free male Sprague-Dawley rats (*n* = 18, 250–280 g; SCXK 2020-0018) were purchased from Liaoning Changsheng Biotechnology Co. (Benxi, China). Specific pathogen-free male Kunming mice (*n* = 108, 20-25 g; SCXK 2015-0018) were purchased from Hubei Experimental Animal Research Center (Wuhan, China). The animals were allowed to acclimatize for 1 week before they were used for experiments, and were maintained under standard laboratory conditions of 24°C ± 2°C on a 12:12-h light/dark cycle with free access to food and water for the duration of the study. All the experiments and animal care were performed strictly in accordance with the Provision and General Recommendation of Chinese Experimental Animals Administration Legislation, and were approved by the Animal Ethics Committee of Hubei University of Chinese Medicine (No: syxk 2017-0067).

### Preparation of PA, PMRP, and ATR Aqueous Extracts

250 g PMRP and 250 g ATR were mixed and put into a decoction bag. The mixture was soaked in distilled water for 1 h, and then extracted twice with reflux extraction method. It was extracted for 2 h with distilled water (1:10, w/v) for the first time and for 1 h with distilled water (1:8, w/v) for the second time. The extract solution was filtered and concentrated to the concentration of the drug 1.56 g dry material/mL. The solution was separated and stored at −20 C.

250 g PMRP were put into a decoction bag and soaked in distilled water for 1 h. It was extracted twice with reflux extraction method, 2 h with distilled water (1:10, w/v) for the first time and 1 h with distilled water (1:8, w/v) for the second time. The extract solution was filtered and concentrated to the concentration of the drug 0.78 g dry material/mL. The solution was separated and stored at −20 C.

250 g ATR were put into a decoction bag and soaked in distilled water for 1 h. It was extracted twice with reflux extraction method, 2 h with distilled water (1:10, w/v) for the first time and 1 h with distilled water (1:8, w/v) for the second time. The extract solution was filtered and concentrated to the concentration of the drug 0.78 g dry material/mL. The solution was separated and stored at −20 C.

The doses of PA, PMRP, and ATR were calculated according to body surface area and were expressed as Gram of the original dry material per kilogram body weight. The oral dosages for mice corresponded to the human dosage in the Chinese Pharmacopoeia (Chinese Pharmacopoeia Commission, 2015).

### Animal Treatment

The mice were randomly divided into the following eight groups (*n* = 12 per group): 1) control (normal feeding); 2) model [scopolamine, 4 mg kg^−1^⋅day^−1^ by intraperitoneal injection (i.p.)]; 3) PAL [scopolamine, 4 mg kg^−1^⋅day^−1^ i. p. + PA, 1.56 g kg^−1^⋅day^−1^ by intragastric administration (i.g.)]; 4) PAH (scopolamine, 4 mg kg^−1^⋅day^−1^ i. p. + PA, 6.24 g kg^−1^⋅day^−1^ i. g.); 5) PL (scopolamine, 4 mg kg^−1^⋅day^−1^ i. p. + PMRP, 0.78 g kg^−1^⋅day^−1^ i. g.); 6) PH (scopolamine, 4 mg kg^−1^⋅day^−1^ i. p. + PMRP 3.12 g kg^−1^⋅day^−1^ i. g.); 7) AL (scopolamine, 4 mg kg^−1^⋅day^−1^ i. p.+ ATR, 0.78 g kg^−1^⋅day^−1^ i. g.); and 8) AH (scopolamine, 4 mg kg^−1^⋅day^−1^ i. p. + ATR, 3.12 g kg^−1^⋅day^−1^ i. g.). Mice in each group were orally administered PA, PMRP, or ATR every day for 30 days; after the last gavage, scopolamine was intraperitoneally injected and the Morris water maze experiment was conducted 40 min later for 6 days.

Additionally, 18 rats were orally administered PA, PMRP, or ATR once, respectively (6 rats in each group). The doses were 4.32 g/kg for PA, 2.16 g/kg for PMRP or ATR. The whole blood samples were collected at different time points.

### HPLC-MS Analysis of PA

#### The Profile Comparison Between PA and PMPR + ATR

PMRP and ATR were mixed 1:1 after separately extracted as a group of (PMRP + ATR). The difference of components in PA and PMRP + ATR was investigated. The samples were separated on a Eclipse Plus C_8_ RRHD-phase column (2.1 × 100 mm, 1.8 μm) (Agilent Technologies Co., Ltd., United States). The autosampler was set at 20 C, and the gradient elution was employed with 0.1% formic acid as solvent A and acetonitrile as solvent B. The gradient program was used as follows: 0-13 min, 95%–68% A; 13-29 min, 68%–1% A; 29–30 min, 1%–95% A. The flow rate was set at 0.3 ml min^−1^, and the injection volume was 2 μL. The total run time was 30 min for each sample.

#### Analysis of Components in PA

Standard stock solutions (0.1 mg/ml) of four components (i.e. THSG, gallic acid, emodin-8-O-β-d-glucopyranoside, and emodin), and Ibuprofen were prepared individually by dissolving an appropriate amount of each chemical standard in a known volume of methanol. The standard stock solutions prepared were kept at −20 C when not used. The standard working solutions of the four components and Ibuprofen were prepared individually by diluting each stock solution with methanol.

The components of PA were analyzed by HPLC-MS. The samples were separated on a ZORBAX StableBond C_18_ reversed-phase column (2.1 × 50 mm, 1.8 μm) (Agilent Technologies Co., CA, United States). The autosampler was set at 20 C, and gradient elution was performed with 0.1% formic acid as solvent A and acetonitrile as solvent B. The gradient program was as follows: 0–10 min, 88%–40% A; 10–12 min, 40%–1% A; and 12–15 min, 1% A. The flow rate was set at 0.3 ml min^−1^, and the injection volume was 4 μL. The total run time was 15 min for each sample.

### GC-MS Analysis of PA

#### The Profile Comparison Between PA and PMPR + ATR

GC-MS analysis was conducted on ThermoFisher Trace 2000 DSQ with Quadrupole MS Filter (Thermo Fisher Scientific). The carrier gas was 99.99% high-purity helium at a flow rate of 1.0 ml/min. Samples were separated on a TR-5MS column (30 × 0.25 mm and 0.25 μm film thickness). The oven temperature program was initially set at 50°C for 2 min, then ramped at 5°C/min to 180°C and held for 5 min; ramped at 20°C/min to 240°C and held for 2 min. The total run time was 38 min.

#### Analysis of Components in PA

Standard stock solutions (0.1 mg/ml) of four components (i.e. β-asarone, α-asarone, *cis*-methyl isoeugenol, and asarylaldehyde), and Naphthalene were prepared individually by dissolving an appropriate amount of each chemical standard in a known volume of methanol. The standard stock solutions prepared were kept at −20°C when not used. The standard working solutions of the four components and Naphthalene were prepared individually by diluting each stock solution with methanol.

GC-MS analysis was conducted on a 7890A series gas chromatograph coupled to a Model 7,693 autosampler and Model 5975C Inert XL EI MSD with Triple-Axis Detector (Thermo Fisher Scientific). The carrier gas was 99.99% high-purity helium at a flow rate of 1.2 ml/min. Samples were separated on a TG-5MS column (60 × 0.25 mm and 0.25 μm film thickness). The sample volume was 1 μL with a split ratio of 5:1. The oven temperature program was initially set at 100°C for 1 min, then ramped at 10°C/min to 180°C and held for 1 min; ramped at 5°C/min to 210°C and held for 1 min; and ramped at 10°C/min to 260°C and held for 5 min. The total run time was 27 min. The injection port and detector temperatures were 280°C and 300°C, respectively. Electrospray ionization was performed at 70 eV. Selective ion monitoring was set for quantitation with a dwell time of 100 m/ion.

HPLC-MS and GC-MS analysis of plasma PA, PMRP, and ATR after oral administration.

Male rats (*n* = 18) were fasted but had free access to water for 12 h prior to administration of PA, PMRP, and ATR (6 rats in each group). Blood samples were collected from the posterior orbital venous plexus of rats before dosing (time 0) and 5, 10, and 30 min and 1, 2, 4, 8, 16, and 24 h after dosing. The samples were immediately centrifuged at 4,500 rpm for 10 min and the separated plasma samples were stored at −80°C until analysis.

### Morris Water Maze Test

Spatial memory was assessed with the Morris water maze test as previously described Lan et al. (2012), with slight modifications. The maze consisted of a black circular pool (120 cm in diameter and 60 cm in height) with a featureless inner surface. A round escape platform was placed 1 cm below the water surface in the center of one quadrant. Each mouse was tested twice a day for 5 days and the latency to reach the platform was recorded. When the mouse found the platform, it was allowed to stay on it for 10 s; if the mouse failed to find the platform within 90 s, it was guided there and allowed to stay on it for 15 s, and the escape latency was recorded as 90 s. On day 6, the platform was removed and the mouse was allowed to swim freely for 90 s as the probe test. The time that the mouse spent in the target quadrant (ie, where the platform was previously hidden) and the number of times it crossed the platform were measured. The point of entry of the mouse into the pool and location of the platform for escape was the same in trials 1 and 2 but was changed each day thereafter.

### HPLC-MS Analysis of Neurotransmitter Content in Mouse Cortical Tissue

Six mice per group were deeply anesthetized with chloral hydrate (320 mg/kg, i. p.) 60 min after the behavioral test and perfused through the ascending aorta with phosphate-buffered saline (PBS). The brains were removed immediately in 0.9% cold saline on a cold plate. Then, the cortical tissue was dissected and weighed. Cortical tissue samples from mice (50 mg) were placed in Eppendorf tubes and pure water was added at 2 times the volume; after homogenization for 2 min, 3 times the volume of precipitant was added to the tube (1:1 methanol:acetonitrile), followed by vortexing for 2 min and centrifugation for 10 min. The supernatant was aspirated, and 5 μL of the sample was injected into the HPLC-MS system with an Acclaim TM RSLC Explosives E2 column (2.1 × 150 mm, 2.2 μm) (Thermo Fisher Scientific). The autosampler was set at 35°C, and gradient elution was performed with 0.1% formic acid as solvent A and methanol as solvent B. The gradient program was as follows: 0–2 min, 96% A; 2–5 min, 96%–20% A; 5–6 min, 20%–1% A; 6–7 min, 1%–96% A. The flow rate was set at 0.2 ml min^−1^, and the injection volume was 4 μL. The total run time was 10 min for each sample.

### Western Blot Analysis

Three mice per group were deeply anesthetized with chloral hydrate (320 mg/kg i. p.) 60 min after the behavioral test and perfused through the ascending aorta with PBS. The brains were removed immediately in 0.9% cold saline on a cold plate. Then, the hippocampus tissue was dissected and weighed. The hippocampus tissue was lysed in radioimmunoprecipitation assay buffer containing protease and phosphatase inhibitors; the protein concentration of the lysate was determined with the Bradford assay. Proteins were separated by SDS-PAGE and transferred to a polyvinylidene difluoride membrane. After blocking with 5% bovine serum albumin for 1 h, the membrane was incubated overnight at 4°C with antibodies against BDNF, TrkB, ERK, *p*-ERK, CREB, *p*-CREB, and actin (all at 1:5,000 dilution). After three washes with washing buffer and incubation with secondary antibody (1:3,000) for 2 h, protein bands were detected with SuperSignal West Pico Chemiluminescent Substrate (Solarbio, Beijing, China) and the signal intensity of target protein bands was normalized to that of the loading control (actin).

### Histology and Immunohistochemistry

Three mice per group were deeply anesthetized with chloral hydrate (320 mg/kg i. p.) and perfused through the ascending aorta with saline solution followed by 4% paraformaldehyde in PBS. The brain was removed and immediately fixed in 4% paraformaldehyde in PBS. After 24 h, the brain was dehydrated and embedded in paraffin blocks, and cut into 4-μm sections. After quenching endogenous peroxidase and blocking with normal goat serum, the sections were incubated overnight at 4°C with antibodies against P90RSK and PSD95 (both at 1:5,000). The sections were washed with PBS and incubated with secondary antibodies for 2 h at 37°C, and then stained with 3,3′-diaminobenzidine in chromogen solution; they were then counterstained with hematoxylin, dehydrated in ethanol, cleared in xylene, and mounted with Cytoseal (Thermo Fisher Scientific). P90RSK and PSD95 immunoreactivity was measured using Image Pro Plus v6.0 software (Media Cybernetics, Rockville, MD, United States). Brown staining on the cell membrane or in the cytoplasm was represented positive staining, with the staining intensity reflecting the expression levels of P90RSK and PSD95 proteins.

### Statistical Analysis

All experiments were performed at least three times. Data for Latency and time in each quadrant in behavior test are expressed as mean ± S.D. and were analyzed by two-way ANOVA followed by Bonferroni test. All other data are expressed as mean ± S.D. and were analyzed by one-way ANOVA followed by Bonferroni test; a *p* value <0.05 was considered significant. Data analysis was performed using Prism v8.0 software (GraphPad, San Diego, CA, United States).

## Results

### HPLC-MS and GC-MS Analysis of PA

**FIGURE 2:**
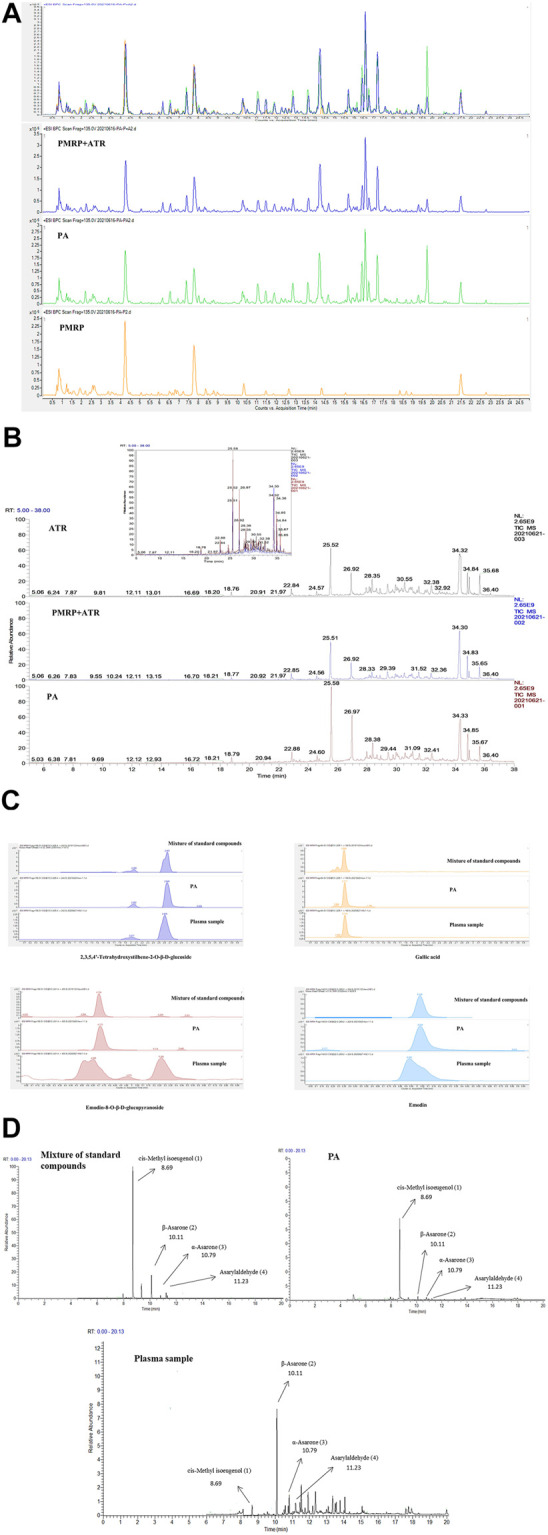
Analysis of PA components.(**A**, **C**) Components of PA analyzed by HPLC-MS.(**B**, **D**) Components of PA analyzed by GC-MS. (1) *cis*-Methyl isoeugenol; (2) α-asarone; (3) β-asarone; and (4) asarylaldehyde.

As shown in Figures 2A, B profile comparison between PA and PMRP + ATR (1:1) was performed by HPLC-MS (2A) and GC-MS (2B). It was shown that there was no obvious difference in the components between PA and PMRP + ATR. Response signals of some components in PA were stronger than those in PMRP + ATR, indicating that the dissolution of some components was enhanced after co-extraction.

Moreover, eight compounds including THSG, gallic acid, emodin-8-O-β-d-glucopyranoside, emodin, β-asarone, α-asarone, *cis*-methyl isoeugenol, and asarylaldehyde were identified in PA by HPLC-MS and GC-MS (Figures 2C, D).

### HPLC-MS and GC-MS Analysis of PA, PMRP, and ATR in Plasma Following Oral Administration

**FIGURE 3:**
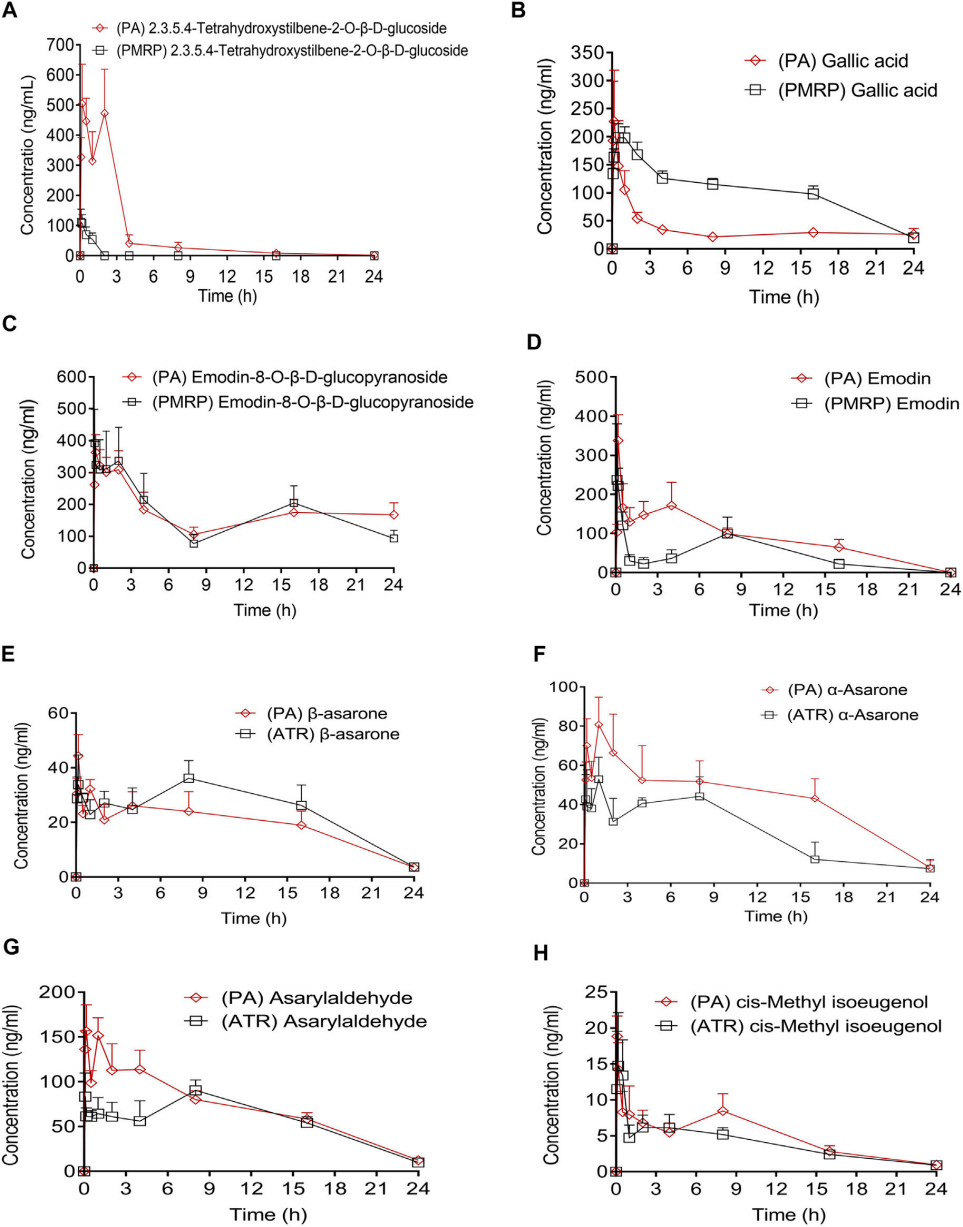
Plasma concentration–time curves of eight compounds in rats following administration of PA, PMRP, and ATR. Data represent mean ± S.D. (*n* = 6).

The main pharmacokinetic parameters including half-time (t_1/2_), maximum plasma concentration (C_max_), time to reach the maximum concentration (T_max_), and area under concentration–time curve (AUC_0−t_ and AUC_0−1_) are shown in Table 1; and plasma concentration–time curves of the eight analytes detected in the rats are shown in Figure 3. THSG and gallic acid were rapidly absorbed following oral administration of PMRP; the T_max_ values were 0.39 and 0.69 h, respectively. In contrast, emodin and emodin-8-O-β-D-glucopyranoside were absorbed slowly with T_max_ values of 2.76 and 5.63 h, respectively. THSG and emodin-8-O-β-D-glucopyranoside were absorbed slowly (T_max_ = 0.81 and 9.58 h, respectively) while gallic acid and emodin were more rapidly absorbed (T_max_ = 0.44 and 0.47 h, respectively) after PA administration. Moreover, β-asarone, α-asarone, *cis*-methyl isoeugenol, and asarylaldehyde were absorbed slowly after oral administration of ATR (T_max_ = 4.78, 3.54, 3.51, and 2.21 h, respectively) and rapidly after administration of PA (T_max_ 0.35, 0.88, 1.58, and 0.43 h, respectively). Plasma concentrations of THSG, emodin, α-asarone, and asarylaldehyde increased after oral administration of PA compared to the levels after administration PMRP or ATR, whereas the concentrations of gallic acid, emodin-8-O-β-d-glucopyranoside, β-asarone, and *cis*-methyl isoeugenol were similar with PA, PMRP, and ATR administration.

**TABLE 1:** Pharmacokinetic parameters in rats following administration of PMRP, ATR, and PA Data represent mean ± S.D. (*n* = 6).

Compounds	C_max_ (ng/mL)	T_max_(h)	T_1/2_(h)	AUC_0-t_ (ng h/mL)	AUC_0-∞_(ng h/ml)
(A)					
2,3,5,4′-Tetrahydroxystilbene-2-O-β-glucoside (in PMRP)	147.404 ± 104.8	0.389 ± 0.356	0.17 ± 0.136	101.501 ± 49.387	102.1 ± 49.699
2,3,5,4′-Tetrahydroxystilbene-2-O-β-glucoside (in PA)	708 ± 280.665	0.806 ± 0.695	3.011 ± 3.358	1,517.388 ± 972.634	1,626.592 ± 1,118.459
Gallic acid (in PMRP)	215.514 ± 47.948	0.694 ± 0.356	6.979 ± 2.149	2,457.058 ± 606.988	2,696.61 ± 822.156
Gallic acid (in PA)	245.502 ± 244.307	0.444 ± 0.763	19.532 ± 13.369	830.982 ± 496.569	1,532.964 ± 898.547
Emodin-8-O-β-D-Glucopyranoside (in PMRP)	490.663 ± 277.498	5.625 ± 8.043	9.261 ± 3.48	5,258.814 ± 2,831.91	10,658.893 ± 6,148.592
Emodin-8-O-β-D-Glucopyranoside (in PA)	420.384 ± 142.541	9.583 ± 7.513	8.25 ± 4.831	5,669.086 ± 2018.946	14,503.234 ± 4,387.838
Emodin (in PMRP)	370.452 ± 287.6	2.764 ± 4.056	3.701 ± 6.831	846.971 ± 801.984	7,398.429 ± 14,360.974
Emodin (in PA)	363.348 ± 121.915	0.472 ± 0.748	14.042 ± 11.818	1808.837 ± 934.333	4,160.976 ± 3,813.521
(B)						
*cis*-Methyl isoeugenol (in ATR)	19.432 ± 18.798	3.514 ± 3.753	7.493 ± 2.55	93.824 ± 20.379	123.263 ± 30.025	
*cis*-Methyl isoeugenol (in PA)	24.126 ± 4.987	1.583 ± 3.163	8.097 ± 2.69	117.609 ± 45.24	129.017 ± 45.513	
β-Asarone (in ATR)	49.752 ± 16.049	4.778 ± 3.777	10.662 ± 6.942	603.44 ± 280.969	1,045.247 ± 910.63	
β-Asarone (in PA)	48.21517.6 ± 9	0.347 ± 0.351	14.015 ± 14.847	439.412 ± 208.854	743.79 ± 620.279	
α-Asarone (in ATR)	73.456 ± 25.933	3.542 ± 3.736	11.797 ± 12.574	599.674 ± 360.384	1,042.098 ± 614.716	
α-Asarone (in PA)	100.899 ± 30.292	0.875 ± 0.699	8.264 ± 2.704	1,017.925 ± 592.53	1,244.69 ± 562.296	
Asarylaldehyde (in ATR)	119.288 ± 52.23	2.208 ± 3.217	7.177 ± 1.232	1,439.382 ± 492.857	1,547.283 ± 482.098	
Asarylaldehyde (in PA)	200.368 ± 44.956	0.431 ± 0.442	7.375 ± 2.935	1706.93 ± 502.851	1968.705 ± 765.551	

### Effect of PA on Spatial Learning and Memory

**FIGURE 4:**
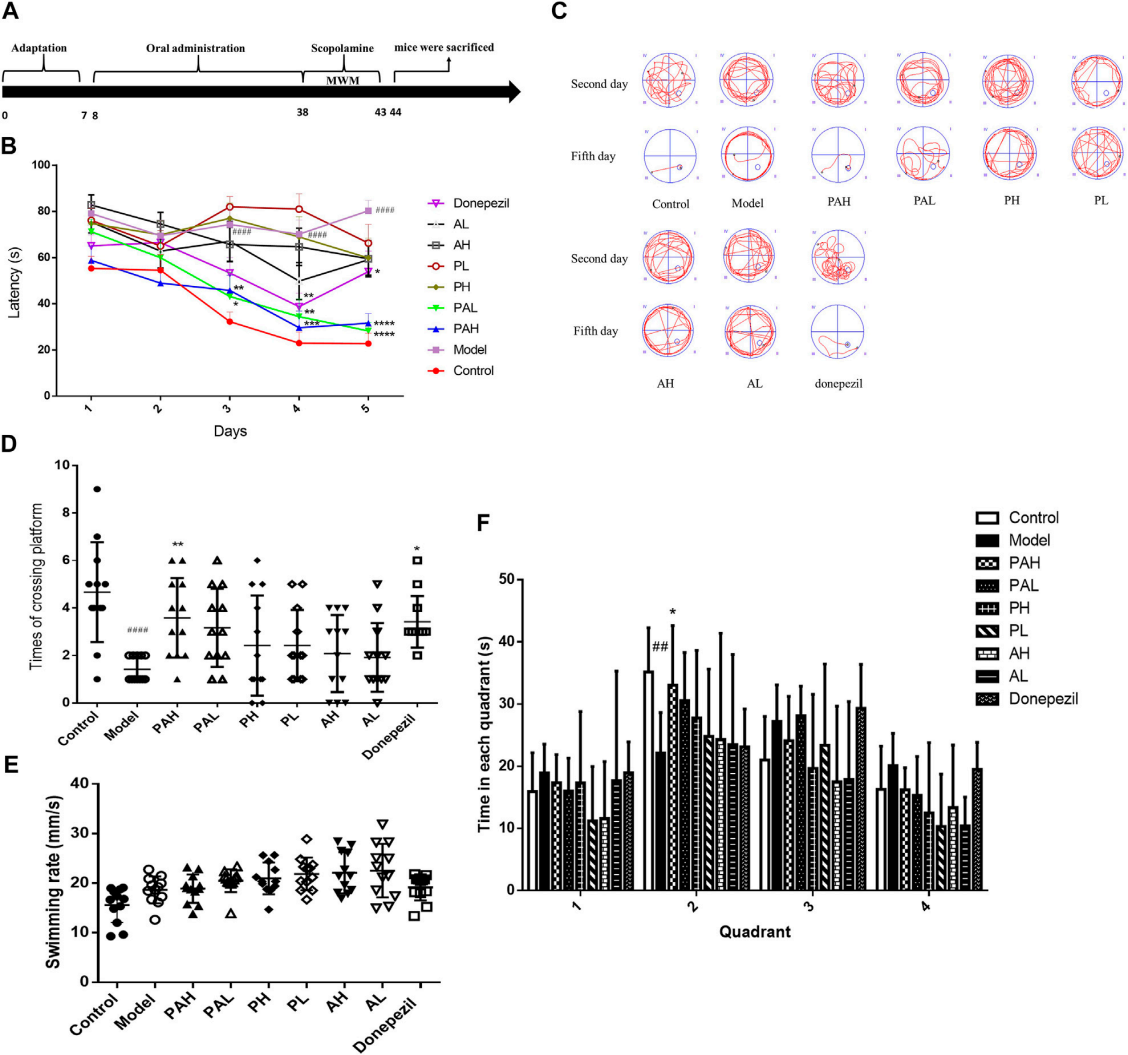
Spatial learning and memory ability of mice as evaluated with the Morris water maze test. (**A**) Experimental schedule of PA administration in scopolamine-treated mice.(**B**) Latency to find a hidden platform in the water maze over five consecutive days of training.(**C**) Representative search strategy of mice on days 2 and 5. Traces show the swimming paths of each group of mice.(**D**) Number of platform crossing in 90 s. E. Swimming rate of each group of mice. F. Time to the different quadrant in the probe test. Data represent mean ± S.D. (*n* = 12). ^*##*^ *p* < 0.01, ^*####*^ *p* < 0.0001 vs control mice; ^*^ *p* < 0.05, ^**^ *p* < 0.01, ^***^ *p* < 0.001, and ^****^ *p* < 0.001 vs scopolamine-treated mice.

Spatial learning and memory of the mice was assessed with the Morris water maze test. The mean latency to find the platform decreased over the five training days (Figure 4A). Scopolamine-treated (ie, dementia model) mice required a significantly longer time to find the platform than controls starting from day 3 (*p* < 0.001), indicating cognitive impairment. Moreover, the increase in escape latency was reversed by treatment with PA (1.56 and 6.24 g/kg) from day 3–5 and by donepezil treatment from day 5 (*p* < 0.05). The swimming paths of mice on days 2 and 5 of the test are shown in Figure 4B. All mice explored the four quadrants of the pool on day 2 but on day 5, control mice swam in the direction of the platform while those treated with scopolamine had longer swimming paths. In the probe test (Figure 4D), control mice required more time to cross the platform than those treated with scopolamine. Additionally, compared to the scopolamine-treated group, mice in the PA (6.24 g/kg) and donepezil treatment groups had more platform crossings. Figure 4F showed that control mice and PAH mice spent more time in the quadrant once the platform placed than that of scopolamine-treated mice. Finally, no significant differences in the swimming rate for mice in each group were observed (Figure 4E), suggesting that treatment with PA, PMRP or ATR had no effect on motor function and motivation.

### HPLC-MS Analysis of Neurotransmitter Content in the Cerebral Cortex of Mice

**FIGURE 5:**
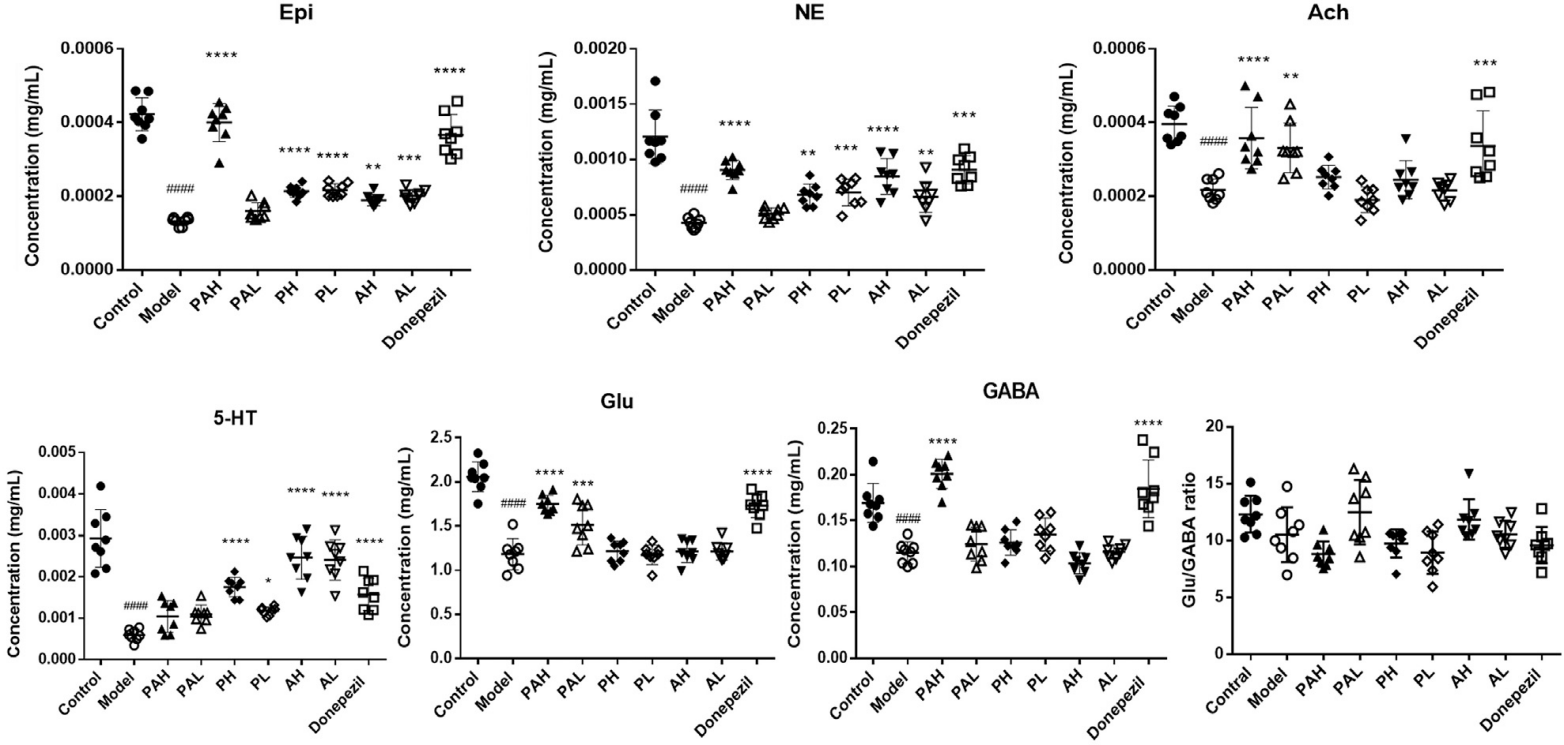
Effect of PA on neurotransmitter levels in scopolamine-treated mice. Data represent mean ± S.D. (*n* = 8). ^*####*^ *p* < 0.0001 vs control mice; ^*^ *p* < 0.05, ^**^ *p* < 0.01, ^***^ *p* < 0.001, and ^****^ *p* < 0.001 vs scopolamine-treated mice.

Scopolamine injection reduced the concentrations of ACh, 5-hydroxytryptamine (5-HT), norepinephrine (NE), epinephrine (Epi), glutamate (Glu), and γ-aminobutyric acid (GABA) in the cortex (Figure 5). ACh and Glu concentrations in the donepezil and PA (1.56 g/kg and 6.24 g/kg) groups but not in the PMRP and ATR groups were significantly higher than that in the scopolamine-treated group (*p* < 0.05). Administration of PA (6.24 g/kg), PMRP (0.78 and 3.12 g/kg), ATR (0.78 and 3.12 g/kg), and donepezil increased the concentrations of the Epi and NE neurotransmitters (*p* < 0.05). Only the highest and lowest doses of PA did not increase the content of 5-HT (*p* < 0.05), and the GABA concentration did not differ among groups except in mice treated with PA (6.24 g/kg) or donepezil. Furthermore, the ratios of Glu/GABA were observed while there were no significant differences among groups.

### Effect of PA on Expression of BDNF/TrkB/ERK/CREB Signaling Axis Components in the Hippocampus

**FIGURE 6:**
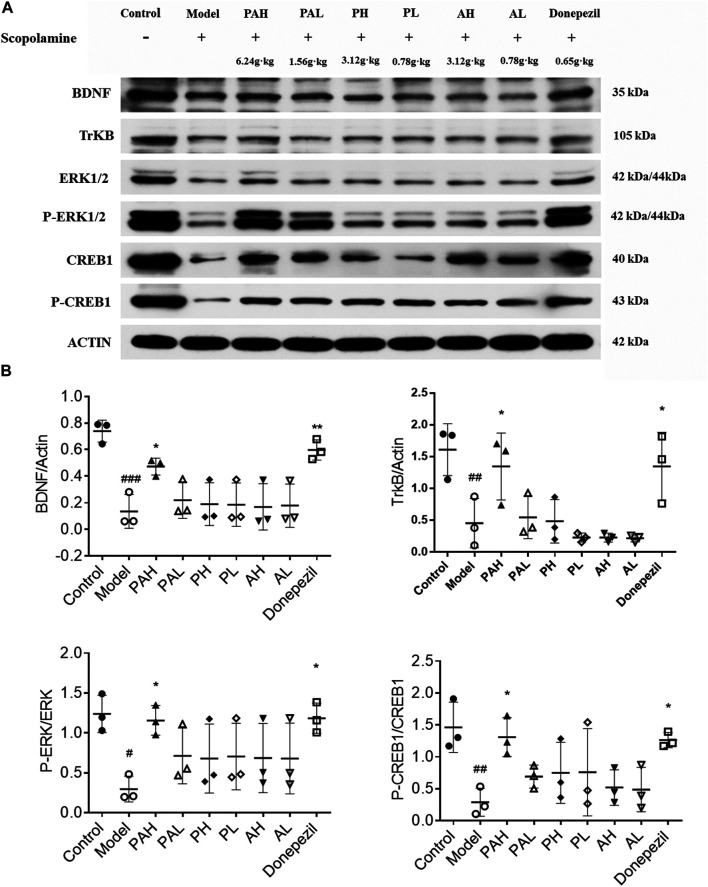
Effect of PA on synaptic protein expression levels in scopolamine-treated mice. Data represent mean ± S.D. (*n* = 3). ^*#*^ *p* < 0.05, ^*##*^ *p* < 0.01 and ^*###*^ *p* < 0.001 vs control mice; ^*^ *p* < 0.05 and ^**^ *p* < 0.01 vs scopolamine-treated mice.

The expression of BDNF, TrkB, ERK, *p*-ERK, CREB, and *p*-CREB proteins in the hippocampus of mice was evaluated by western blotting (Figure 6). Scopolamine treatment reduced the levels of these proteins; this was reversed by administration of the highest doses of PA and donepezil (*p* < 0.05). PMRP and ATR had a similar effect but the results did not reach statistical significance.

### Effect of PA on the Expression of P90RSK and PSD95 Proteins

**FIGURE 7:**
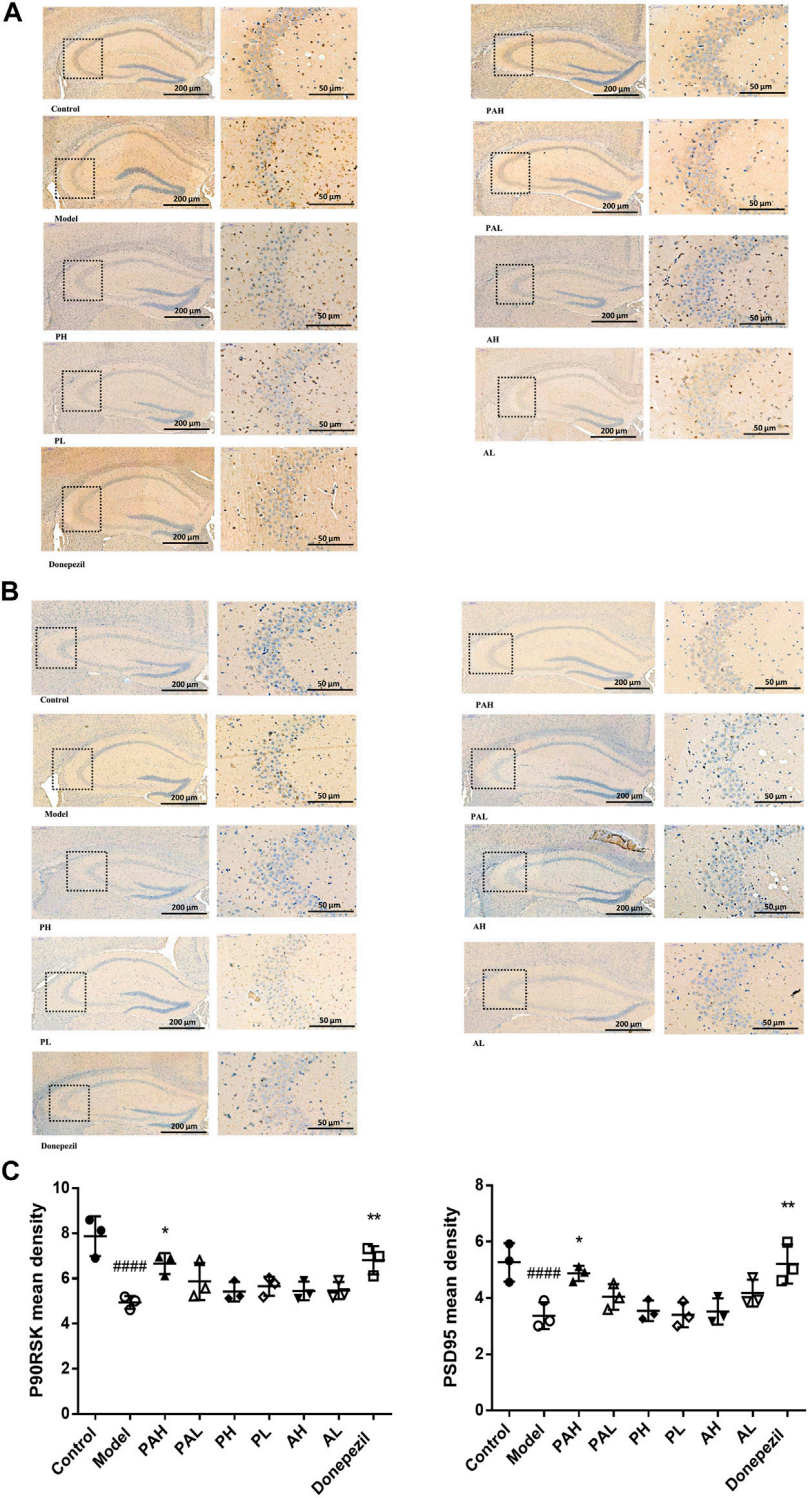
Immunohistochemical analysis of P90RSK and PSD95 in the hippocampus of mice. (**A**, **B**) Expression of P90RSK **(A)** and PSD95 **(B)** in the hippocampus of mice analyzed by immunohistochemistry. C, Mean density of P90RSK and PSD95 determined by Image Pro Plus. Data represent mean ± S.D. (*n* = 3). ^*####*^ *p* < 0.0001 vs control mice; ^*^ *p* < 0.05, **p < 0.01 vs scopolamine-treated mice.

P90RSK (Figure 7A) and PSD95 (Figure 7B) expression in the hippocampus of scopolamine-treated mice was analyzed by immunohistochemistry. Scopolamine reduced the immunoreactivity of these proteins, but this effect was mitigated by administration of PA, PMRP, ATR, and donepezil. High doses of PA and donepezil had a more potent effect than low doses of PA, PMRP, and ATR.

## Discussion

In TCM, AD is known as a disease of forgetfulness. Tang Rongchuan stated in the book of “Essence of Chinese and Western Huitong Medical Classics” that the reason why things are not forgotten depends on the memory, and memory is recorded in the kidney meridian. In the Qing Dynasty, Wang Ang once said in “Ben Cao Bei Yao” that memories are in the brain. If the kidney essence is deficient, the brain will not be replenished to reinforce memory, and the function of the five internal organs will be reduced; the weak infusion of the essence, Qi, blood, and body fluid results in phlegm production, which obstructs memory. Thus, the TCM principle of “invigorating kidney and resolving phlegm” is applied to the prevention of aging-related diseases such as AD that have memory impairment as a symptom (Fu, 2004; Li and Tang, 2006; Wang et al., 2008; Yuan et al., 2008; Cui and Zhang, 2015). PMRP can tonify the kidney and ATR can dissipate phlegm. PMRP had a broad range of pharmacologic activities including anti-aging and neuroprotective effects Li et al. (2005), Kim et al. (2013), Niu et al. (2014), and ATR can improve memory and cognitive function (Deng et al., 2015). Based on these findings above-mentioned, the present study investigated the molecular basis for the synergistic protective effects of PA against memory impairment in aging-related diseases such as dementia.

Scopolamine treatment has been used to model learning and memory disorders including AD in animals (Flood and Cherkin, 1986). Scopolamine is a nonselective muscarinic receptor antagonist that impairs learning and memory function, especially short-term memory and learning acquisition (Beatty et al., 1986). It also has been demonstrated that one of the subtypes of muscarinic receptor, M1 receptor, is most abundant in the forebrain and hippocampus, and specifically involved in memory processes (Levey et al., 1991; Wei et al., 1994; Anagnostaras et al., 2003). More and more researches have focused on the effects of scopolamine treatment on cognitive impairment in the animal’s hippocamus Lee et al. (2018), Corpuz et al. (2019), Garabadu and Sharma (2019), Hernández-Rodríguez et al. (2020), and show that muscarinic activity induced by scopolamine in hippocampus and entorhinal cortex is crucial for spatial and fear memory retrieval (Falsafi et al., 2012; Rashid and Ahmed, 2019). In this study, we used scopolamine-treated mice to explore the effect of PA on learning and memory and found that scopolamine impaired cognitive function as determined by time in the quadrants and number of platform crossings in the Morris water maze test, which is consistent with previous studies (Ionita et al., 2018; Kim et al., 2020). Notably, while PA significantly shortened escape latency from day 3 onwards and increased the number of platform crossings in scopolamine-treated mice, indicating cognitive enhancement, the effects of PMRP and ATR administered individually were not statistically significant, indicating that the 2 drugs act synergistically to reverse scopolamine-induced memory deficits. However, although scopolamine-induced acute amnesia has been proposed as a classical animal model for learning and memory disorders including AD, it is not sufficient to fully interpret the role of PA in the theory of Chinese medicine for invigorating the kidney and resolving phlegm only based on the impairment of learning and memory. The dosage and days of use of scopolamine were determined according to the results of preliminary experiments, which are also the main factors affecting the results. More experiments need to be conducted to clarify the effect of PA on the improvement of cognitive dysfunction with other transgenic disease model.

Changes in neurotransmitter levels in the brain are linked to the development of neurodegenerative diseases. Neurotransmitters are usually stored in the synaptic vesicles, beneath the membrane in the axon terminal, and are released into the synapse with the appropriate signal. Ach, one of excitatory neurotransmitters, plays a vital role in learning and memory consolidation in the brain. Researchers have found that a decrease in AChE activity and, in turn, a decrease in ACh synthesis and uptake, and an eventual loss of memory in AD (Slotkin et al., 1990; Xu et al., 2012). 5-HT is also involved in cognitive mechanisms such as learning and short- and long-term memory in cortex and hippocampus (Cifariello et al., 2008). Concentration of 5-HT are significantly reduced in several brain areas in patients in AD. Scopolamine administration reduced the concentrations of ACh and 5-HT. PA treatment restored the ACh level while PMRP and ATR administered alone increased the concentration of 5-HT. GABA is a neuroinhibitory neurotransmitter, but the precise role of GABA neurotransmitters in AD is not well understood. Postmortem AD patients’ brains have shown significant increases in astrocytic GABA and MAOB expression. Previous study determined that GABA from reactive astrocytes impairs memory in APP/PS1 mouse models (Rissman et al., 2007; Jo et al., 2014). Glu is an ionic form of glutamic acid which is involved in the learning and memory process in the cortex and hippocampus. An increased release of Glu or a failure in re-uptake of released Glu will lead to a tonic activation of NMDA receptors. Glu/GABA ratio has been used as marker of neurochemical brain balance (Revett et al., 2013; Amorini et al., 2017). The ratios were observed in the present study while there were no significant differences among groups, indicating PA, PMRP or ATR had no obvious effects on the ratio of Glu/GABA in the cortex of scopolamine-treated mice. NE and Epi have a role in causing oxidative stress and have been reported to be involved in synaptic activities (Kandimalla and Hemachandra Reddy, 2017). Treatments of PA, PMRP and ATR increased the concentrations of Epi and NE in scopolamine-treated mice.

To clarify the molecular mechanisms underlying the protective effect of PA against cognitive impairment, the expression of memory-related proteins were examined by western blotting and immunohistochemistry in scopolamine-treated mice with or without administration of PA, PMRP, and ATR. BDNF is involved in synaptic plasticity and memory formation (Bekinschtein et al., 2007); BDNF mediates regulation of excitatory synapses and binds to the TrkB receptor to activate a downstream signaling cascade that includes ERK1/2, thereby modulating synaptogenesis and cognitive function. CREB plays a critical role in the formation and consolidation of spatial learning and memory, and is activated through phosphorylation by the upregulation of ERK (Vaynman et al., 2004; Lesiak et al., 2013). For example, activation ERK coupled to CREB is required for BDNF-induced LTP at DG synapse *in vivo* (Ying et al., 2002). Increased expression of BDNF, *p*-ERK, and *p*-CREB has been shown to alleviate cognitive dysfunction. This study found that at a high concentration, PA increased the levels of BDNF, TrkB, *p*-ERK, and *p*-CREB that were reduced by scopolamine, whereas PMRP and ATR alone had little effect. These results suggest that PA improves cognitive dysfunction induced by scopolamine via the BDNF/ERK/CREB signaling pathway. The expression of the synaptic proteins PSD95 and P90RSK was also examined by immunohistochemistry. PSD95 regulates synaptic strength and activity-dependent synaptic plasticity Béïque and Andrade (2003), and p90RSK is a downstream effector of the ERK signaling cascade that is involved in neuroplasticity and synapse formation (Frodin and Gammeltoft, 1999; Pereira et al., 2012). The present study found that P90RSK and PSD95 protein levels were downregulated by scopolamine, but PA administration reversed this effect.

The above results provide evidence that PA alleviates learning and memory impairment in scopolamine-treated mice by modulating synaptic-related proteins. Hence, the precise components of PA that are involved remain to be determined. According to the characteristics of TCM treatment based on syndrome differentiation, Chinese herbal medicine co-extracted decoction is the main form of clinical prevention and treatment of TCM. Given that PA is the product of co-extraction of PMRP and ATR, it is very likely that new compounds could be formed during the process of co-extraction. To clarify this concern, a profile comparison between PA and PMRP + ATR (1:1 mixed, separately extracted) was performed. Components analysis was showed that there was no obvious difference in the components between PA and PMRP + ATR. Response signals of some components in PA were stronger than those in PMRP + ATR, indicating that the dissolution of some components was enhanced after co-extraction. Additionally, eight active compounds in PA and in rat plasma following administration of PA were identified; plasma concentrations of THSG, emodin, α-asarone, and asarylaldehyde were increased by PA but not by PMRP and ATR, whereas gallic acid, emodin-8-O-β-d-glucopyranoside, β-asarone, and *cis*-methyl isoeugenol concentrations were similar with all three formulations. These results demonstrated that the combination use of PMRP and ATR might enhance the absorption of THSG, emodin, α-asarone, and asarylaldehyde. In the present study, rats were received the same original material concentration of PA, PMRP and ATR, and the samples were collected and analyzed at the same times, but the concentrations of each component were not controlled when the rats were orally administrated the extracts, which would bring uncertainty when analyzing differences in the plasma concentration of compounds. It is also important to know whether the synergistic effect is in extraction or in the body. Therefore, additional studies, including comparing the effect of PMRP extract plus ATR extract with PA, controlling the concentration of compounds in PA or PMRP or ATR, pharmacokinetic study of the compound etc., are needed to further determine whether a true synergistic effect exist or not.

It was previously reported that THSG enhanced memory by modulating ERK1/2 and CREB activation and increasing BDNF and PSD95 levels, which had a protective effect on synaptic structure and function (Hou et al., 2011; Wang et al., 2011; Zhou et al., 2012; Shen et al., 2015; Chen et al., 2016). Additionally, emodin protected against synaptic impairment by modulating the ERK1/2/nuclear factor erythroid 2-related factor (Nrf)2/heme oxygenase (HO)-1 pathway Lai et al. (2020) while α-asarone improved learning and memory deficits (Limón et al., 2009; Shin et al., 2014; Li et al., 2019; Chen et al., 2020). The results reveal that THSG, emodin, and α-asarone are the main active components of PA that protect scopolamine-treated mice from cognitive dysfunction, and that PA has greater benefits than PMRP and ATR alone.

In summary, the present study provides evidence for the preventive effect of PA against scopolmine-induced memory deficits in mice. The main findings of the study are specific as follows. First, traditional combination of PA can significantly improve the learning and memory ability of scopolamine-treated mice. Second, administration of PA can increase neurotransmitter levels, induce the activation of the BDNF/ERK/CREB signaling pathway, and regulate the proteins (including p90RSK, PSD95) related to synaptic plasticity in the hippocampus of scopolamine-treated mice. Third, the contents of THSG, emodin, α-asarone, and asarylaldehyde in plasma after PA administration were increased compared to those after PMRP or ATR administration, suggesting that these four compounds might be the main active components contributed to the combination effect. Thus, the traditional combined use of PMRP and ATR might achieve a synergistic effect in the treatment of dementia, and embodies the principle of “invigorating the kidney and resolving phlegm” of TCM. The present findings highlight the therapeutic potential of PA for the treatment of aging-related diseases including AD.

## Data Availability

The original contributions presented in the study are included in the article/supplementary material, further inquiries can be directed to the corresponding authors.
